# Coupled Anisotropic Magneto-Mechanical Material Model for Structured Magnetoactive Materials

**DOI:** 10.3390/polym12112710

**Published:** 2020-11-16

**Authors:** Eike Dohmen, Benjamin Kraus

**Affiliations:** Institute of Mechatronic Engineering, Chair of Magnetofluiddynamics, Measuring and Automation Technology, Technische Universität Dresden, 01062 Dresden, Germany; benjamin.kraus@tu-dresden.de

**Keywords:** magneto-active materials, MR elastomers, anisotropic, magneto-mechanical, material model, coupled approach

## Abstract

Adaptability of properties of magnetic materials such as magnetorheological (MR) fluids, MR elastomers (MRE), and other magneto-active (MA) materials drives scientific activities worldwide, trying to broaden the fields of application of such materials. In our work, we focused on the utilization and implementation of existing material models to realize a praxis-oriented coupled anisotropic material model for the commercial finite element (FE) software ABAQUS taking into account magneto-mechanical interactions. By introducing this material model, a first step is done to predict and optimize the behavior of MA materials.

**Data Set License:** CC-BY

## 1. Introduction

Suspensions of magnetic soft microparticles with carrier fluids are usually referred to as magnetorheological (MR) fluids, suspensions of single domain nanoparticles as ferrofluids. If particles are distributed in a cross-linked elastomer matrix, the resulting compound is called MR elastomer. An externally applied magnetic field provides remote control of such composite materials’ physical properties, where these are often grouped under the generic term magneto-active (MA) materials. Due to their adaptability, this material group is of high interest for numerous technical applications. However, since many of these applications involve complex magnetic fields with strongly position-dependent field strengths and field orientations, MA materials will be affected by the field, resulting in locally varying material behavior and locally varying deformations [1,2,3,4,5,6,7], which in turn influence the magnetic field. Therefore, for an efficient utilization of MA materials, advanced material models are necessary, capable of describing the dependency of rheological or mechanical properties on field strength, temperature, load type, load direction, and field orientation. Although there are many scientific investigations on these materials, the interdependence of magnetic field orientation, load direction, and mechanical properties is still unsatisfactory taken into account [8]. Certainly, this is due to the complex interaction of a magnetic field with mechanical stresses and deformations of such materials and vice versa, i.e., the influence of deformation and the material’s internal magnetic structure on the magnetic field.

### 1.1. Magnetically Influenced Anisotropy

The behavior of MR fluids is significantly affected by an external magnetic field due to the formation of e.g., rod-like or net-like superstructures [9,10,11,12,13,14]. Though the mechanisms are different for MR elastomers due to the fixation of magnetic particles within the elastomeric matrix, particle movement can be observed [15]. The endeavor of magnetic particles to arrange energetically favorable in superstructures aligned with the direction of the magnetic field and the deformation of these superstructures due to mechanical stresses is similar to both, MR fluids as well as MR elastomers or more general to MA materials and therefore needs to be considered within simulations. For MR elastomers, field orientation may differ from the orientation of particle superstructures as it is possible to manufacture pre-structured specimen by applying a magnetic field during crosslinking of the elastomeric matrix. Due to this, pre-structured MR elastomers have a preferential magnetic direction which is fixed to the polymer network and in contrast to MR fluids show an anisotropic off-state material behavior in the absence of a magnetic field. When a magnetic field is applied, this anisotropic material behavior will be changed depending on the magnetic field direction relative to the pre-structuring direction. With regard to a praxis-oriented theoretical description of both isotropic MA materials and anisotropic pre-structured variants, it is necessary to provide a versatile material model which may be adapted easily based on experimental results and is capable of estimating or predicting MA materials’ behavior. This is especially true for complex assemblies with MA materials where field orientation is highly dependent on the assemblies kinematic state.

### 1.2. Existing Material Theories

Theoretical description and simulation of MA materials was performed by many work groups with different goals [16,17,18,19,20,21]. However, to simulate complex assemblies and get a deeper understanding of experimentally observed effects, it is necessary to find an appropriate theoretical description for such magneto-mechanical coupled anisotropic MA materials, where a distinction between material’s anisotropic preferential direction and field direction is possible. In the relevant literature, a variety of different approaches can be found. Kalina et al. [22] and Ivanekyo et al. [23] give a detailed overview of existing theories and models for MR elastomers, of which two selected approaches—one based on microscopic scale interactions and one based on macroscopic phenomena described by continuum mechanics—shall be briefly introduced in the following.

An example for the microscopic approach are particle interaction approaches as presented by Jolly et al. [24], Han et al. [25], and Ivaneyko et al. [23]. Here, the magnetic filler particles are approximated as dipoles. In doing so, it is possible to calculate a simplified interaction energy to estimate the material behavior of MR elastomers. Nevertheless, this assumption is only valid for dilute systems, where the distance between two separate particles is large compared to their diameter. Additionally, this approach assumes a quasi-static homogeneous magnetic field, although the local magnetic field changes with deformation. Therefore, Biller et al. [26] proposed an extension of this approach, utilizing multipoles and therefore being more suitable for materials with a higher volume fraction of magnetic filler particles.

The macroscopic approach, on the other hand, considers the material as a homogeneous composite and couples magnetic field equations with mechanic field equations to describe the macroscopic material behavior. Therefore, the material is abstracted as a homogeneous continuum. This approach is presented by Bustamante [27,28,29,30], Dorfmann and Ogden [31] as well as Brigadnov et al. [32].

## 2. Modeling Approach

For experimentally investigating MA materials magnetic field orientation dependent behavior, a variety of measuring devices are known [33,34,35,36,37,38,39,40]. As pre-structured MR elastomers depict all of the relevant aspects needed to verify a suitable model, we will focus on these in our work, as it will be possible to transfer our findings to other MA materials.

### 2.1. Magneto-Mechanical Coupling

Aligning with the idea of implementing and providing a praxis-oriented material model for Finite Element (FE) simulations, the macroscopic continuum mechanics based approach is used hereafter unless otherwise stated, as this approach enables an analysis of more complex real life assemblies based on a praxis-oriented abstraction level, dealing with different parts and materials, while merging microscopic details into one continuous material. For the realization of an appropriate magneto-mechanical anisotropic material model, the renowned commercial FE software package ABAQUS by Dassault Systèmes^®^ (Vélizy-Villacoublay, France) is used, offering a fully documented interface for user material (UMAT) subroutines. The striven coupling of magnetic and mechanical fields leads to constitutive equations which describe the physical behavior of real materials under defined boundary conditions [41].

For a successful implementation of the material model into a UMAT subroutine, it is necessary to derive the tangent stiffness matrix from the constitutive equations.

### 2.2. Kinematics

**Figure 1:**
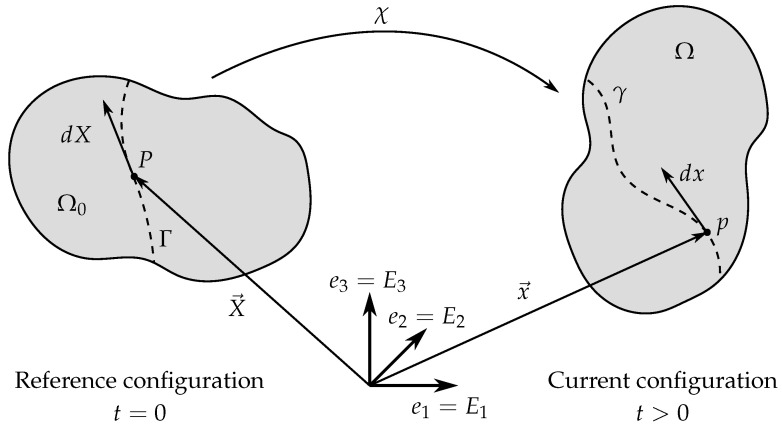
Visualization of different descriptive approaches for the movement and deformation of a continuum body in space by two selected configurations in time (t=0 reference configuration, t>0 current configuration). Describing a movement or deformation relative to the coordinates of a reference configuration (undeformed) is called Lagrangian description, while describing it relative to the coordinates of a current configuration (deformed) is called Eulerian description (adapted from [41]).

Distortions and deformations are induced due to the movement of a material body and can be described by kinematics. As illustrated in Figure 1, the movement $\vec{\chi }$ can be defined with regard to a reference configuration (Lagrangian description) or with regard to the current configuration (Eulerian description).

All variables with regard to the current configuration will be marked lower case, while all variables with regard to the reference configuration will be represented by upper case letters or by the index *l*. Figure 1 shows the reference configuration (t=0) and the actual configuration (t>0) of a body. Within this work, both conditions relate to the same Cartesian coordinate system, where the basis point for the reference configuration following the Lagrangian description is the same as for the current configuration following the Eulerian description. The coordinate system is defined as Cartesian, with E=e=I as unit matrix.

In our reference configuration, a point *P* is defined by its material coordinates vector $\vec{X}$ at the time t=0 and is part of section $\Omega_{0}$. At a different time t>0, this section $\Omega_{0}$ is transformed due to a deformation into a section Ω. *P* is defined by the spatial coordinates vector $\vec{x}=\vec{\chi }\left(\vec{X},t\right)={\left[x_{1},x_{2},x_{3}\right]}^{T}$. Additionally, an undeformed line Γ is observed, on which the point *P* is located. As a result of a movement $\vec{\chi }$, this line is deformed to a spatial line γ. The associated physical or rather spatial tangent vectors are defined as $d\vec{X}$ and $d\vec{x}$ as done by Holzapfel [41]. With these two tangent vectors, the deformation gradient F is defined as partial derivative of the movement $\vec{\chi }$ with respect to material coordinates vector $\vec{X}$:

(1) $$F=\frac{d\vec{x}}{d\vec{X}}=\frac{\partial \vec{\chi }\left(\vec{X},t\right)}{\partial \vec{X}},$$

being a measure for the deformation in nonlinear continuum mechanics. Its determinant can be calculated from that

(2) J=det(F)>0

This determinant is always positive and for incompressible media defined as J≡1. Furthermore, with the deformation gradient F, both the left and right Cauchy-Green deformation tensor

(3) $$b=F\;\cdot \;F^{T}$$

and

(4) $$C=F^{T}\;\cdot \;F$$

may be calculated. Both of them are necessary for a description of the Helmholtz free energy density function Ω(F)=Ω(C)=Ω(b).

### 2.3. Mechanical Stress Definition

Deformation and distortion build up tension in the material. To describe the tension in the material, we define the Cauchy stress tensor as:

(5) $$\left[\sigma \right]=\left[\begin{array}{ccc} \sigma_{11} & \tau_{12} & \tau_{13}\\ \tau_{21} & \sigma_{22} & \tau_{23}\\ \tau_{31} & \tau_{32} & \sigma_{33} \end{array}\right]$$

At the principal diagonal, the normal stresses $\sigma_{ij}$ are defined and the shear stresses $\tau_{ij}$ are at the secondary diagonals. For the numerical algorithm, we need the Kirchhoff stress tensor

(6) τ=Jσ

which corresponds to the Cauchy stress tensor for incompressible materials. In continuum mechanics, it is most often necessary to define the stress tensor in relation to a reference section. This stress tensor with dependence on τ is referred to as the first Piola–Kirchhoff stress

(7) $$P=\tau F^{-T}$$

Due to its definition, the first Piola-Kirchhoff stress is asymmetrical and therefore more complex to use for the continuum stress definition. For this reason, the first Piola-Kirchhoff stress tensor can be transferred to the second Piola–Kirchhoff stress

(8) $$S=F^{-1}\tau F^{-T}$$

Although the second Piola-Kirchhoff stress can not be directly interpreted physically, it has the advantage of being symmetrical and being defined in the initial configuration. Hence, the second Piola-Kirchhoff stress tensor is more suitable for FEM simulations.

### 2.4. Magnetic Field Equations

Additional to the stress definition, it is necessary for characterization of MR elastomers to know the equations for the effect of the magnetic field. Assuming a linear relationship between magnetic flux density $\vec{B}$ and magnetic field strength $\vec{H}$, the relationship is given by

(9) $$\vec{B}=\mu_{0}\;\left(\vec{H}\;+\;\vec{M}\right)=\mu \;\vec{H}$$

where $\mu_{0}$ is the magnetic constant—being the magnetic permeability for vacuum—and μ is the magnetic permeability. To take care regarding a magnetically transversal isotropic material behavior, different magnetic permeabilities may be considered. To consider the relationship between magnetic fields and mechanical stresses, the Maxwell stress tensor ${Ø}_{\mathrm{maxwell}}$ is utilized:

(10) $$\tau_{\mathrm{maxwell}}=\left[\vec{H}⊗\mu \;\vec{H}-\frac{1}{2}\;\vec{H}\;\cdot \;\mu \;\vec{H}\right)\;I\;].$$

### 2.5. Total Stress Tensor

In the present work, the behavior of the magnetorheological elastomer is described by a hyper-elastic transversely isotropic material model based on publications by Bustamante [28,29] and Dorfmann et al. [30,42]. Aligning to this and to link the magnetic and mechanical properties of the material, the total stress for our approach is composed of mechanical and Maxwell stresses:

(11) $$\tau =\tau_{\mathrm{mechanical}}+\tau_{\mathrm{maxwell}}$$

Bustamante [28,29] and Dorfmann et al. [30,42] define the total stress tensor in dependence on the Helmholtz free energy Ω. Hence, for a compressible material, the total stress tensors follow as

(12) $$\begin{array}{cc} \tau & =J^{-1}\;F\;\frac{\partial \Omega }{\partial F} \end{array}$$

(13) $$\begin{array}{cc} S & =2\;\frac{\partial \Omega }{\partial C} \end{array}$$

and for an incompressible material:

(14) $$\begin{array}{cc} \tau & =F\;\frac{\partial \Omega }{\partial F}\;-\;p\;I \end{array}$$

(15) $$\begin{array}{cc} S & =2\;\frac{\partial \Omega }{C}\;-\;p\;C^{-1}. \end{array}$$

For the hyper-elastic transversely isotropic material, the approach of Bustamante [29] describes the Helmholtz free energy density function

(16) $$\Omega =\Omega (I_{1},I_{2},I_{3},I_{4},I_{5},I_{6},I_{7},I_{8},I_{9},I_{10})$$

depends on 10 invariants—thus combining the magneto mechanical coupling and a preferred direction

(17) $$\vec{a}=F\;\vec{a_{0}}.$$

$\vec{a_{0}}$ is used to control pre-structuring or superstructure (“chain”) direction, where $a_{x}=0$, $a_{y}=0$ and $a_{z}=0$ is for the isotropic case and $a_{x}=0$, $a_{y}=0$ and $a_{z}=1$ is one possibility for a transverse isotropic case.

The invariants mentioned are defined as follows in [29]:

(18) $$\begin{array}{cccccc} I_{1} & =\mathrm{tr}(C) & I_{2} & =\frac{1}{2}\;\left[{\left(\mathrm{tr}\left(C\right)\right)}^{2}-\mathrm{tr}\;\left(C^{2}\right)\right] & I_{3} & =\det\;(C)\\ I_{4} & =\vec{H_{l}}\;\cdot \;\vec{H_{l}} & I_{5} & =\vec{H_{l}}\;\cdot \;C\;\vec{H_{l}} & I_{6} & =\vec{H_{l}}\;\cdot \;C^{2}\;\vec{H_{l}}\\ I_{7} & =\vec{a_{0}}\;\cdot \;C\;\vec{a_{0}} & I_{8} & =\vec{a_{0}}\;\cdot \;C^{2}\;\vec{a_{0}} & I_{9} & =\vec{a_{0}}\;\cdot \;\vec{H_{l}}\\ I_{10} & =\vec{a_{0}}\;\cdot \;C\;\vec{H_{l}} \end{array}$$

where $\vec{H_{l}}=F\;\vec{H}$.

A nearly incompressible material is assumed for the simulation to be carried out. Hence, the deformation gradient is to be decomposed into a volume-preserving (isochoric) part and a volume-changing (dilatational) part. The decomposition is based on the following relation by Ogden [43]

(19) $$\begin{array}{cc} F & =F^{\mathrm{vol}}\;\cdot \;F^{\mathrm{iso}} \end{array}$$

(20) $$\begin{array}{cc} F^{\mathrm{vol}} & =J^{1/3}\;I \end{array}$$

(21) $$\begin{array}{cc} F^{\mathrm{iso}} & =\bar{F}=J^{-1/3}\;F \end{array}$$

$\bar{F}$ specifies the isochoric part of the deformation gradient and thus applies $\det\;\bar{F}=1$ [30]. With the decomposed deformation gradient, isochoric parts of the left and right Cauchy-Green deformation tensors follow to

(22) $$\begin{array}{cc} \bar{b} & =\bar{F}\;{\bar{F}}^{T}=J^{-2/3}\;b \end{array}$$

(23) $$\begin{array}{cc} \bar{C} & ={\bar{F}}^{T}\;\bar{F}=J^{-2/3}\;C \end{array}$$

Thereby, the mentioned invariants can be defined in reference to the decomposed deformation tensor as

(24) $$\begin{array}{cccccc} \bar{I_{1}} & =J^{-2/3}\;I_{1} & \bar{I_{2}} & =J^{-4/3}\;I_{2} & \bar{I_{3}} & =1 \end{array}$$

(25) $$\begin{array}{cccccc} \bar{I_{4}} & =I_{4} & \bar{I_{5}} & =J^{-2/3}\;I_{5} & \bar{I_{6}} & =J^{-4/3}\;I_{6} \end{array}$$

(26) $$\begin{array}{cccccccc} \bar{I_{7}} & =J^{-2/3}\;I_{7} & \bar{I_{8}} & =J^{-4/3}\;I_{8} & \bar{I_{9}} & =I_{9} & \bar{I_{10}} & =J^{-2/3}\;I_{10} \end{array}$$

From the modified invariants, a modified Helmholtz free energy function follows, which can be decomposed into an isochoric $\Omega_{\mathrm{iso}}$ and a volumetric part $\Omega_{\mathrm{vol}}$.

(27) $$\Omega =\Omega_{\mathrm{iso}}\left(I_{1},I_{2},I_{4},I_{5},I_{6},I_{7},I_{8},I_{9},I_{10}\right)+\Omega_{\mathrm{vol}}\left(J\right)+\Omega_{0}$$

As Bustamente [29] already noted, this function has too many free parameters to determine reliability by an experiment. For this reason, not all invariants should be used. For the matrix material, we assume a neo-Hookean elastomer, so that $I_{2}$ can be eliminated. As the invariant $I_{6}$ describes the correlation between the same parameters as $I_{5}$ (just with different exponents), $I_{6}$ is neglected assuming small deformations. The same is true for $I_{8}$ and $I_{7}$, which is why $I_{8}$ is neglected as well. Based on these assumptions and the publications of Bustamante [28,29] and Dorfmann et al. [30,42], the total stress can be defined as:

(28) $$\begin{array}{cc} S & =2\frac{\partial \Omega_{\mathrm{iso}}\left(\bar{C}\right)}{\partial C}\;+\;2\;\frac{\partial \Omega_{\mathrm{vol}}\left(J\right)}{\partial C}=J^{-2/3}\;\mathrm{Dev}\left(\bar{S}\right)\;+\;2\;\frac{\partial \Omega_{\mathrm{vol}}}{\partial J}\;\frac{\partial J}{\partial C}\\ S & =J\;{\bar{\Omega }}_{J}\;C^{-1}+2\;J^{-2/3}\;\left[{\bar{\Omega }}_{1}\;\left(I-\frac{1}{3}\;I_{1}\;C^{-1}\right)+\right.\\ \; & \left.{\bar{\Omega }}_{5}\left(\vec{H_{l}}⊗\vec{H_{l}}-\frac{1}{3}\;\vec{H_{l}}\;\cdot \;C\;\cdot \;\vec{H_{l}}\;\cdot \;C^{-1}\right)+{\bar{\Omega }}_{7}\left(\vec{a_{0}}⊗\vec{a_{0}}-\frac{1}{3}\;\vec{a_{0}}\;\cdot \;C\;\cdot \;\vec{a_{0}}\;\cdot \;C^{-1}\right)+\right.\\ \; & \left.\frac{1}{2}\;{\bar{\Omega }}_{10}\left(\vec{a_{0}}⊗\vec{H_{l}}+\vec{H_{l}}⊗\vec{a_{0}}-\frac{1}{3}\left\{\vec{a_{0}}\;\cdot \;C\;\cdot \;\vec{H_{l}}+\vec{H_{l}}\;\cdot \;C\;\cdot \;\vec{a_{0}}\right\}\;\cdot \;C^{-1}\right)\right]. \end{array}$$

For the further simulation, the total Cauchy stress tensor

(29) $$\begin{array}{cc} \sigma & ={\bar{\Omega }}_{J}I+2\;J^{-5/3}\;\left[{\bar{\Omega }}_{1}\left(b-\frac{1}{3}\;I_{1}\;I\right)+\right.\\ \; & \left.{\bar{\Omega }}_{5}\left(b\;\cdot \;\vec{H}⊗b\;\cdot \;\vec{H}-\frac{1}{3}\;\vec{H_{l}}\;\cdot \;C\;\vec{H_{l}}\;I\right)+{\bar{\Omega }}_{7}\left(\vec{a}⊗\vec{a}-\frac{1}{3}\;\vec{a_{0}}\;\cdot \;C\;\vec{a_{0}}\;I\right)+\right.\\ \; & \left.\frac{1}{2}{\bar{\Omega }}_{10}\left(\vec{a_{0}}⊗b\;\vec{H}+b\;\vec{H}⊗\vec{a_{0}}-\frac{1}{3}\left\{\vec{a_{0}}\;\cdot \;C\;\vec{H_{l}}+\vec{H_{l}}\;\cdot \;C\;\vec{a_{0}}\right\}I\right)\right]. \end{array}$$

is required, where both equations conform to the following conventions:

(30) $$\begin{array}{cccc} {\bar{\Omega }}_{i} & =\frac{\partial \Omega }{\partial {\bar{I}}_{i}} & \; & i=1,5,7,10\\ {\bar{\Omega }}_{J} & =\frac{\partial \Omega }{\partial J} \end{array}$$

For the subsequent implementation, the following equation for the Helmholtz free energy Ω is used as introduced by Bustamante [29,30]):

(31) $$\begin{array}{cc} \Omega & =\left(\frac{{\bar{I}}_{1}-3}{2}\right)\left(g_{0}+g_{1}\;{\bar{I}}_{4}\right)-\ln\left[\cosh(\frac{\sqrt{{\bar{I}}_{4}}}{m_{1}})\right]m_{0}\;m_{1}-\frac{1}{2}\;\zeta_{0}\;{\bar{I}}_{4}+\frac{1}{2}\;\mu_{0}\;{\bar{I}}_{5}\\ \; & +\left(h_{0}+h_{1}\;\ln\left({\bar{I}}_{7}\right)-\frac{h_{1}}{m}\;{\bar{I}}_{7}^{m}\right)\left(\omega_{0}+\omega_{1}\;{\bar{I}}_{9}^{2}+\omega_{2}\;{\bar{I}}_{10}^{2}+\omega_{3}\;{\bar{I}}_{9}\;{\bar{I}}_{10}\right)\\ \; & +m_{0}\;\ln\left[\frac{\cosh^{m_{2}}\left({\bar{I}}_{9}/m_{2}\right)}{\cosh^{m_{1}}\left({\bar{I}}_{9}/m_{1}\right)}\right]+\left(\zeta_{0}-\zeta_{1}-\mu_{0}\right)\frac{{\bar{I}}_{9}^{2}}{2}+\frac{1}{2}\;\kappa {\left(J-1\right)}^{2}+\Omega_{0} \end{array}$$

Referring to Equation (11), the Maxwell share has to be subtracted from the total Cauchy stress tensor to obtain the mechanical stress

(32) $$\tau_{\mathrm{mechanical}}=\tau -\tau_{\mathrm{maxwell}}$$

## 3. Implementation

### 3.1. Utilized Software Package

For the realization of an appropriate magneto-mechanical anisotropic material model, the commercial FE software package ABAQUS is chosen, offering a strong, fully documented interface for user material (UMAT) subroutines and being a praxis-proven FE solution for engineers. Additionally, it is capable of realizing automated coupled simulations via Fortran scripting. Most important for our approach is not only the implementation of existing material theories, but the distinction between material’s anisotropic preferential direction and the actual magnetic field direction. With this, it is possible to define a material direction for every FE element taking into account anisotropic material behavior. Therefore, a field dependent anisotropy may be considered, e.g., describing an off-state anisotropy due to pre-structuring as well as a different on-state state anisotropy induced by a magnetic field or a mechanical load. From this, a material model for commercial FE systems is introduced being capable to describe a complex magneto-mechanical material behavior.

### 3.2. Iterative Coupling Method

As mentioned before, ABAQUS is capable of coupling different simulation types via a Fortran scripting interface—for example magneto-statics with mechanical simulations.

**Figure 2:**
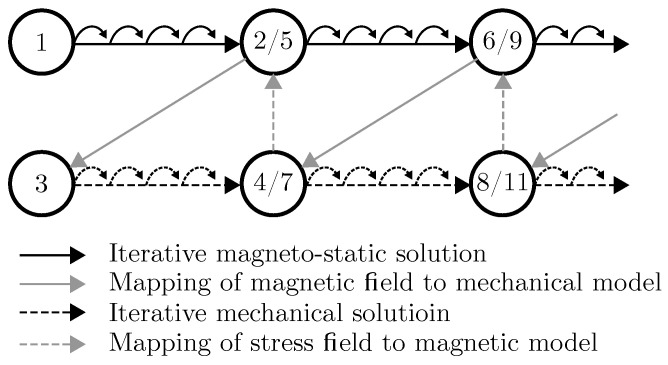
Adapted Conventional Serial Staggered (CSS) Coupling Procedure for the coupling of magneto-static and mechanical simulation; numbers in circles are to be understood as step numbers.

The procedure used within our implementation methodology is a conventional serial staggered coupling procedure illustrated in Figure 2.

Starting with an undeformed but transversely-isotropic reference geometry (step 1), the magnetic field is iteratively calculated until a magneto-static solution is found (step 2) for the undeformed state. This magnetic field configuration is then transferred to the mechanical solver and mapped to the undeformed mesh in (step 3). Now, the mechanical solver iteratively calculates a deformed geometry (step 4) based on loads applied, taking the local, element-wise mechanical material properties into account resulting from:

- element orientation (e.g., corresponding to a pre-structuring direction),
- mapped magnetic field orientation,
- mapped magnetic field strength, and
- defined magneto-mechanical material behavior.

The stress field from the converged mechanical solution is then transferred and mapped to the magneto-static solver (step 5), which calculates a new solution (step 6), which then is transfered back to the mechanical solver (step 7) and so forth. By this coupled iterative CSS approach, a balance between stress field and magnetic field can be approximated. Within this work, one time step ΔT is solely simulated. As described, the magnetic field for the undeformed geometry is simulated first and afterwards the deformation is calculated.

**Figure 3:**
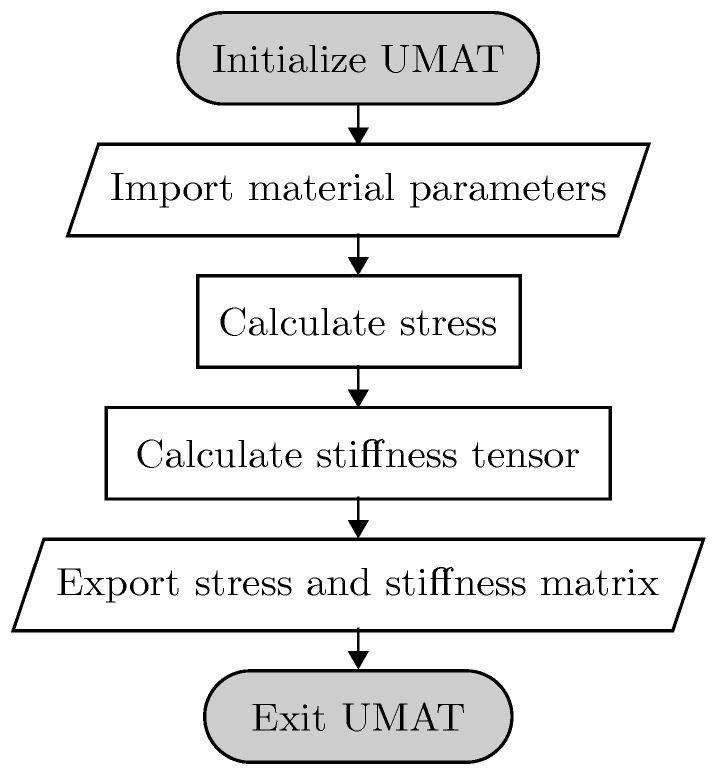
Major sequential substeps in the realized user material subroutine.

To calculate the material behavior of a MR elastomer, a new magneto-mechanical material has to be defined in ABAQUS. To do so, we used the ABAQUS Subroutine UMAT. The basic structure for this subroutine is illustrated in Figure 3.

While solving the FE model, the UMAT will be called by ABAQUS Standard to calculate both the material stress (STRESS) and the associated tangent stiffness (DDSDDE) matrix for each element, depending on the predefined material parameters. The abbreviation DDSDDE is adopted from ABAQUS representing ∂Δσ/∂Δϵ. Within the UMAT, the magneto-mechanical coupled total stress is calculated with the equations presented, while the tangent stiffness matrix is calculated by a numerical algorithm taken from [44]. In connection, STRESS and DDSDDE are returned to ABAQUS Standard to calculate deformation. The generated Fortran script for the Dohmen-Kraus-UMAT is provided as supplementary materials and is licensed under the Creative Commons Attribution 4.0 International License 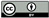.

## 4. Discussion

### 4.1. Simulation

All subsequent simulations for the validation of the developed UMAT and its implementation are performed with a cubic single element of type *C3D8RH* with 1 mm edge length. The UMAT is implemented in Abaqus 2017 by a Fortran script utilizing Visual Studio 2010 (Microsoft Corporation, Redmond, WA, USA) and Fortran Composer 2011 (Intel Corporation, Santa Clara, CA, USA).

To verify the general functionality of the implementation routine of the UMAT, a linear elastic isotropic material behavior as well as a nonlinear neo-Hookean material behavior were implemented as a UMAT. The results were identical to existing material models proving the functionality of the UMAT implementation.

As a next step, the magneto-mechanical transversely-isotropic material model is checked for plausibility by performing simulations with it for isotropic and anisotropic configurations as well as for different field directions and different loading directions. All parameters used for the simulations are summarized in Table 1 and Table 2. κ is neglected as volume-preservation is assumed. Magnetic constant $\mu_{0}$ is $1.2566\times 10^{-6}$ N/kA${}^{2}$. Material parameters were taken from Bustamante et al. [29,30] for deformations below 10% (1≤λ≤1.1). These parameters were obtained by Bustamante fitting experimental data of Bellan and Bossis (see Figure 2 in [45]) for MR elastomers with Φ=15Vol.-% of HQ type carbonyl iron particles from BASF (Ludwigshafen am Rhein, Germany) with an average diameter of 2 μm and a saturation magnetization of $M_{S}=1710\;kA\;m^{-1}$ [45].

The transversally isotropic MRE samples by Bellan et al. were manufactured by pre-structuring during the curing process of the RTV silicone matrix for 10 min at 200 $kA\;m^{-1}$. Experimental measurements in the presence of a magnetic field were performed by Bellan et al. at 123 $kA\;m^{-1}$.

The specified magnetic field directions are given relative to the direction of force application. The mechanical stresses and strains are given for the direction of the force application. As stability issues occurred with anisotropic configurations for magnetic field strengths above 50 $kA\;m^{-1}$, all presented validation results for our UMAT were performed up to magnetic fields of 30 $kA\;m^{-1}$ and for solely one time step.

#### 4.1.1. Isotropic Model

**Figure 4:**
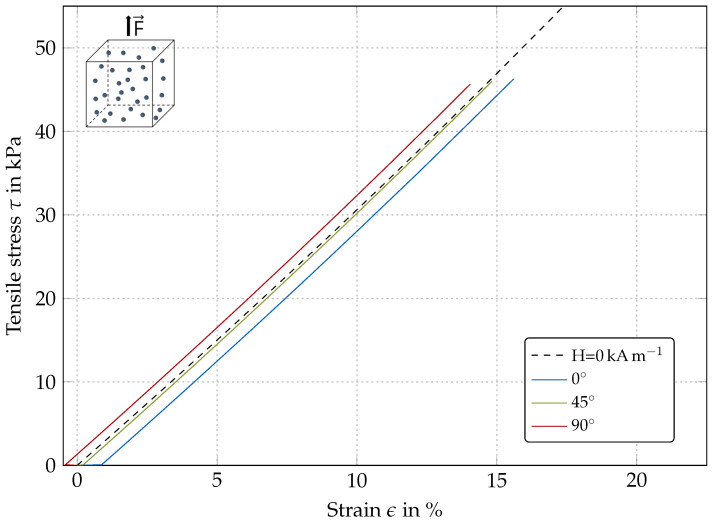
Simulated stress–strain behavior of an isotropic MR elastomer for different magnetic field orientations (H = 30 $kA\;m^{-1}$).

Figure 4 shows the simulation results for an isotropic material for different magnetic field angles.

The stress-strain responses for all field angles are linear within the viewed range for small deformations, which is in accordance with the initial behavior of neo-Hookean law. However, for higher deformations, the material behavior should be nonlinear, but could not be validated within this work due to convergence issues.

No magnetic field: Taking a closer look at the diagram origin, one can see the simulation without a magnetic field (black curve) starting precisely at the origin, whereas the simulations under the influence of a magnetic field are shifted.

Magnetic field at $0^{\circ }$: For the $0^{\circ }$ simulation (blue curve), the magnetic field direction is aligned parallel to the direction of force application, where the model is elongated in the direction of force application as no mechanical load is present. This model is therefore elongated by the magnetic field in addition to the tensile force, which is why the $0^{\circ }$ curve (blue) is shifted to the right. This effect is known as magnetostriction from experimental studies for MA materials, e.g., [46,47,48].

Magnetic field at $90^{\circ }$: Looking at the $90^{\circ }$ results (red curve), the stress–strain curve is shifted upwards relative to the origin. As the magnetic field direction is aligned perpendicular to the direction of force application, the model is elongated in the direction of the magnetic field and at the same time contracts in the direction of force application due to Poisson’s ratios. With increasing tensile force, the initial contraction is overcome.

Magnetic field at $45^{\circ }$: As the effects described for $0^{\circ }$ and $90^{\circ }$ are more or less balanced for $45^{\circ }$, the resulting curve (green) is congruent with the results without a magnetic field.

#### 4.1.2. Transversely-Isotropic Models

**Figure 5:**
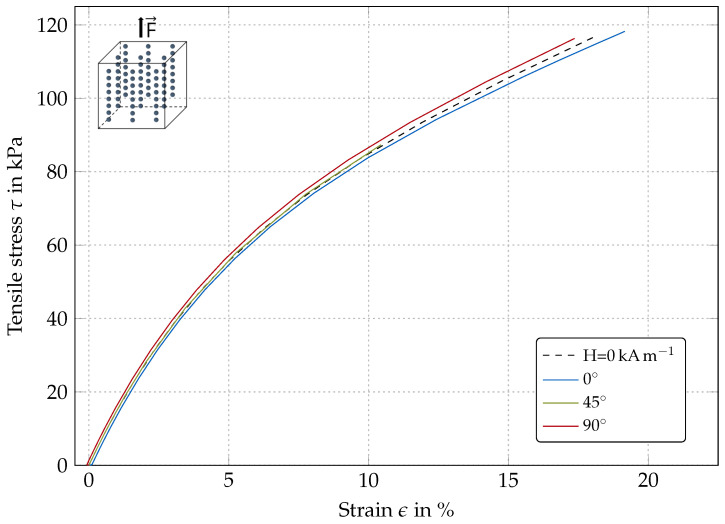
Simulated stress–strain behavior of an anisotropic MR elastomer with pre-structuring directions parallel to direction of force application for different magnetic field orientations (H = 30 $kA\;m^{-1}$).

**Figure 6:**
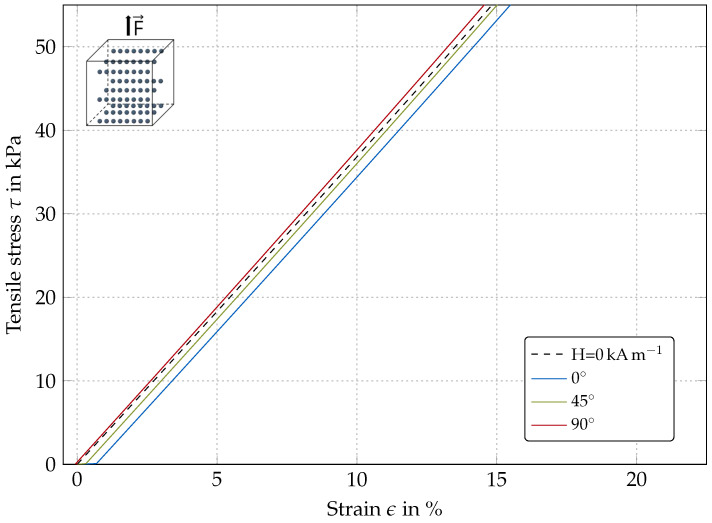
Simulated stress-strain behavior of an anisotropic MR elastomer with pre-structuring directions perpendicular to direction of force application for different magnetic field orientations (H = 30 $kA\;m^{-1}$).

Figure 5 and Figure 6 show the simulation results for a transversely-isotropic material for different magnetic field angles. The results in Figure 5 are for a model with the pre-structuring (particle chain) direction being parallel to the magnetic field direction. The results in Figure 6 are for a model with pre-structuring direction perpendicular to magnetic field direction.

Pre-structuring parallel to load direction: For the material pre-structured parallel to the direction of load, a considerably different behavior as for the isotropic MRE can be seen in Figure 5. Due to the logarithmic approach for Helmholtz free energy density function describing the relation between total stresses and the structure formation, the mechanical stress follows a logarithmic trend. The material is essentially stiffer than both other cases discussed before. The strain initiated by the applied magnetic field is of smaller influence, as the overall off-state stiffness is increased due to the existing superstructures.

Pre-structuring perpendicular to load direction: The behavior for a material pre-structured perpendicular to the direction of load is similar to overall behavior of an isotropic MRE. For the $90^{\circ }$ (red) curve in Figure 6, magnetic field direction is equal to the direction of superstructures, which is why particles are already oriented energetically efficient and the curve is hardly shifted compared to the results without a magnetic field; however, the modulus of elasticity is slightly increased. In contrast, the $0^{\circ }$ simulation (blue curve) in Figure 6 is shifted to the right as the pre-structured particles try to align with the magnetic field initially as no mechanical load is present, where the model is elongated in the direction of force application.

### 4.2. Summary

Our implementation concept was successfully validated enabling praxis-oriented users to realize coupled magneto-mechanical simulations of complex multi-component assemblies with MA materials. The plausibility and general functionality of the implemented material model in the new Dohmen-Kraus-UMAT was shown. The UMAT was used to successfully describe the complex field dependent anisotropic magneto-mechanical behavior of MR elastomers. Nevertheless, due to convergence issues, the considered magnetic field strengths were limited to small values up to H = 30 $kA\;m^{-1}$, where the resulting effects are fairly small. This is also true for the simulated deformations which are also limited to approximately 10% due to convergence stability. As the development of this model was especially done for MR elastomers with higher deformations, this is a major issue for our approach, which we want to address in future works. Additionally, an experimental validation with field orientation dependent stress–strain curves would be of high value for a further analysis of the capabilities of our approach.

## 5. Conclusions

Within this work, a new praxis-oriented anisotropic magneto-mechanical material model for the commercial FE system Abaqus and a corresponding coupled magneto-mechanical simulation approach are presented. The focus of this work is not a new implementation of existing material theories, but an introduction of a magneto-mechanical material model for transversely-isotropic MA materials (e.g., pre-structured MR elastomers) being capable of an element-wise consideration of material direction, magnetic field direction and magnetic field strength. Utilizing the presented Dohmen-Kraus-UMAT, a material’s field dependent anisotropy can be considered, e.g., describing an off-state anisotropy due to pre-structuring as well as a distorted on-state state anisotropy induced by a magnetic field or a mechanical load. From this, simulations of transversely-isotropic MA components and assemblies, which allow mapping of magnetic-field induced changes of both mechanical and magnetic anisotropy, are enabled for the first time with a commercial FE software. Although the presented Dohmen-Kraus-UMAT is fully functional, there are speed and stability issues which are to be addressed in future works.

## Figures and Tables

**Table 1:** Material parameters used for validation taken from [29].

Parameter	Value	Unit	Source
*k*	1		Table 1 in [29]
$g_{0}$	0.1	M Pa	Table 1 in [29]
$g_{1}$	−1	MPa/(kA/mm)${}^{2}$	Table 1 in [29]
$h_{0}$	0.113578		Table 2a in [29]
$h_{1}$	0.007633		Table 2a in [29]
$\omega_{0}$	2.227236	MPa/(kA/mm)${}^{2}$	Table 2a in [29]
$\omega_{1}$	40.182	MPa/(kA/mm)${}^{2}$	Table 2a in [29]
$\omega_{2}$	−7.653	MPa/(kA/mm)${}^{2}$	Table 2a in [29]
$\omega_{3}$	19.688	MPa/(kA/mm)${}^{2}$	Table 2a in [29]
*m*	−10		[29] (p. 197)

**Table 2:** Permeabilities μ used for our magnetostatic simulations; based on initial gradients (up to 50 $kA\;m^{-1}$) from experimental data.

isotropic (no pre-structuring)	3.675
pre-structuring direction parallel to field direction	4.3125
pre-structuring direction perpendicular to field direction	3.589

## Supplementary Materials

The following are available online at https://www.mdpi.com/2073-4360/12/11/2710/s1. The supplementary material is the “Dohmen-Kraus-UMAT.for” Fortran file.

## Abbreviations

The following abbreviations are used in this manuscript:

MR	magnetorheological
MA	magneto-active
FE	finite element
UMAT	ABAQUS user material subroutine
